# Micro-Spherical Sulfur/Graphene Oxide Composite via Spray Drying for High Performance Lithium Sulfur Batteries

**DOI:** 10.3390/nano8010050

**Published:** 2018-01-18

**Authors:** Yuan Tian, Zhenghao Sun, Yongguang Zhang, Xin Wang, Zhumabay Bakenov, Fuxing Yin

**Affiliations:** 1 School of Material Science & Engineering, Research Institute for Energy Equipment Materials, Hebei University of Technology, Tianjin 300130, China; tianyuanhebut@163.com (Y.T.); sunzhenghao666@163.com (Z.S.); 2 Synergy Innovation Institute of GDUT, Heyuan 517000, China; wangxin@scnu.edu.cn; 3 Institute of Batteries LLC, National Laboratory Astana, Nazarbayev University, 53 Kabanbay Batyr Avenue, Astana 010000, Kazakhstan; zbakenov@nu.edu.kz

**Keywords:** sulfur/graphene oxide (S/GO) composites, lithium sulfur batteries, spray drying

## Abstract

An efficient, industry-accepted spray drying method was used to synthesize micro-spherical sulfur/graphene oxide (S/GO) composites as cathode materials within lithium sulfur batteries. The as-designed wrapping of the sulfur-nanoparticles, with wrinkled GO composites, was characterized by scanning electron microscopy (SEM) and transmission electron microscopy (TEM). The unique morphological design of this material enabled superior discharge capacity and cycling performance, demonstrating a high initial discharge capacity of 1400 mAh g^−1^ at 0.1 C. The discharge capacity remained at 828 mAh g^−1^ after 150 cycles. The superior electrochemical performance indicates that the S/GO composite improves electrical conductivity and alleviates the shuttle effect. This study represents the first time such a facile spray drying method has been adopted for lithium sulfur batteries and used in the fabrication of S/GO composites.

## 1. Introduction

Advanced power sources have been commonly employed in the mobile electronic device and hybrid electric vehicle markets [1,2,3,4,5]. Lithium sulfur batteries show great potential for large-scale application in various green energy fields, due to the ultra-high theoretical capacity of sulfur, reaching 1675 mAh g^−1^ [6,7,8,9]. Additionally, sulfur has the advantages of being inexpensive, environmentally benign, and naturally abundant, making it attractive for practical applications [10,11]. However, there are some intrinsic issues that have plagued sulfur cathodes. The low electronic conductivity of sulfur leads to poor electrochemical performance and low utilization during charging and discharging processes. Additionally, the high dissolution of intermediate polysulfides in electrolyte solvents is another important issue to consider when designing long cycle-life sulfur composite electrodes [12]. To overcome the above problems, various types of conductive carbon and conductive polymer have been employed to improve conductivity and limit polysulfide dissolution [13,14,15]. Among them, graphene (a type of carbon material) can provide a highly electrical conductivity network with a large surface area, trapping the dissolved polysulfides via the physical adsorption of graphene. In Kumar et al.’s study, a facile spray drying process to fabricate sulfur/graphene composite was reported, where the ink consisted of sulfur nanoparticles and graphene flakes [16]. The batteries of S/graphene composites as cathode materials show a high discharge capacity of 1500 mAh g^−1^ and excellent cycle properties, with approximately 70% of capacity retained after 250 cycles. Qiu et al. [17] synthesized a nitrogen-doped graphene to wrap sulfur nanoparticles, showing high specific discharge capacity as well as good cycling stability. This indicates that N-doping in graphene could effectively inhibit polysulfide diffusion and reduce polysulfide shuttling. Furthermore, the oxidized derivative of graphene (i.e., graphene oxide (GO)) has received little attention from researchers in the fabrication of sulfur cathode composites for lithium sulfur batteries [18]. The oxygen functional groups on graphene oxide’s edges and basal planes can be used to inhibit polysulfide shuttling [19,20].

Herein, for the first time, we report the use of a commercial spray drying strategy to prepare a micro-spherical sulfur/graphene oxide (S/GO) composite, in which graphene oxide-wrapped sulfur particles serve as traps for sulfur and polysulfide intermediates via both physisorption and chemisorption. Evaluation of the physical and electrochemical properties of the composite as a cathode material for lithium sulfur batteries is presented.

## 2. Results and Discussion

**Figure 1:**
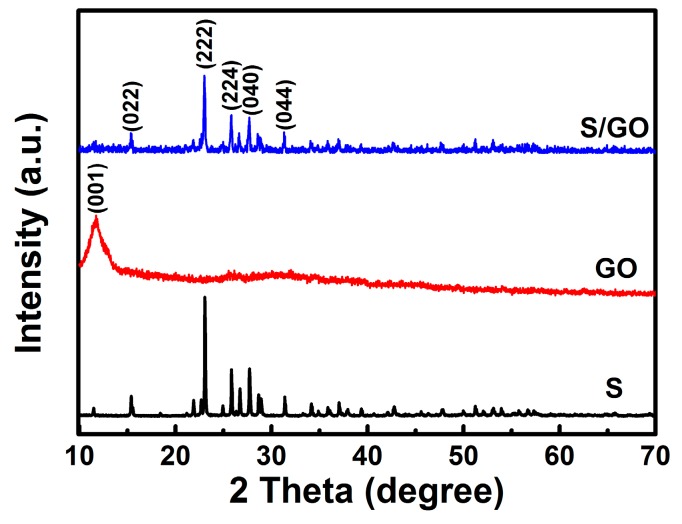
XRD patterns of S, GO, and S/GO composite powders.

The crystal structures of sulfur nanoparticles, GO and S/GO composite powders derived by the spray drying method, were tested using X-ray diffraction (XRD, shown in Figure 1). The XRD spectrum of the S/GO composite revealed orthorhombic sulfur peaks with reduced intensity due to GO. The sulfur nanoparticles exhibited prominent peaks at around 23° and 28° in the S/GO composite powders, which represents the (222) and (040) of sulfur nanoparticle reflections [21]. The detected characteristic diffraction peaks of the sulfur nanoparticles and S/GO composite powders match very well, demonstrating the orthorhombic structure of the sulfur nanoparticles. These results indicate that the sulfur nanoparticles were encapsulated in the S/GO composite powders.

**Figure 2:**
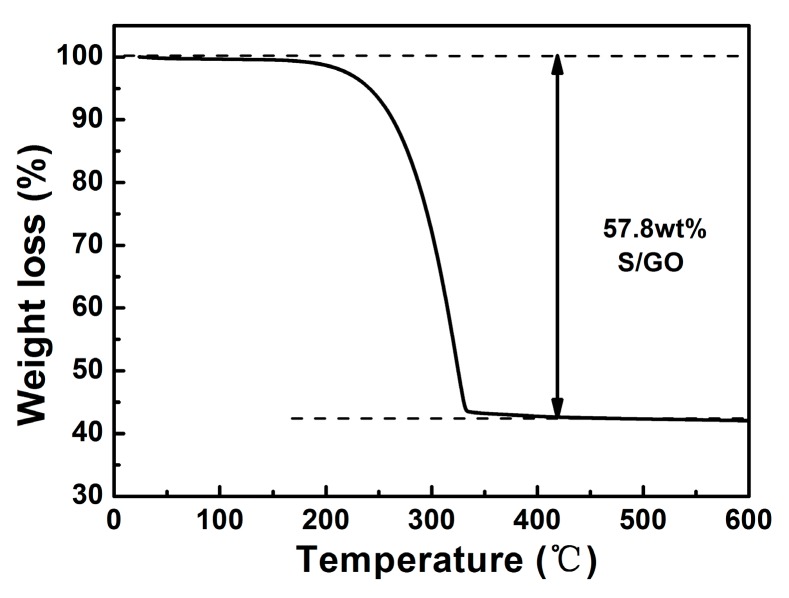
TGA curve of S/GO composite at a heating rate of 10 °C min^−1^.

In order to determine the sulfur content in the S/GO composite, thermogravimetric analysis (TGA) of the sample was conducted in argon, with a heating rate of 10 °C min^−1^, as shown in Figure 2. The sample weight loss occurred in two steps. At the temperature range of 40–170 °C, the sample lost about 6% of its weight, due to the evaporation of water. When the temperature was further increased from 170 °C to 340 °C, rapid weight loss occurred, due to the evaporation of sulfur. When the temperature further increased to 600 °C, the sample weight remained almost unchanged. Therefore, the TGA data allowed for the estimation of sulfur content in the S/GO composite at approximately 57.8 wt %.

**Figure 3:**
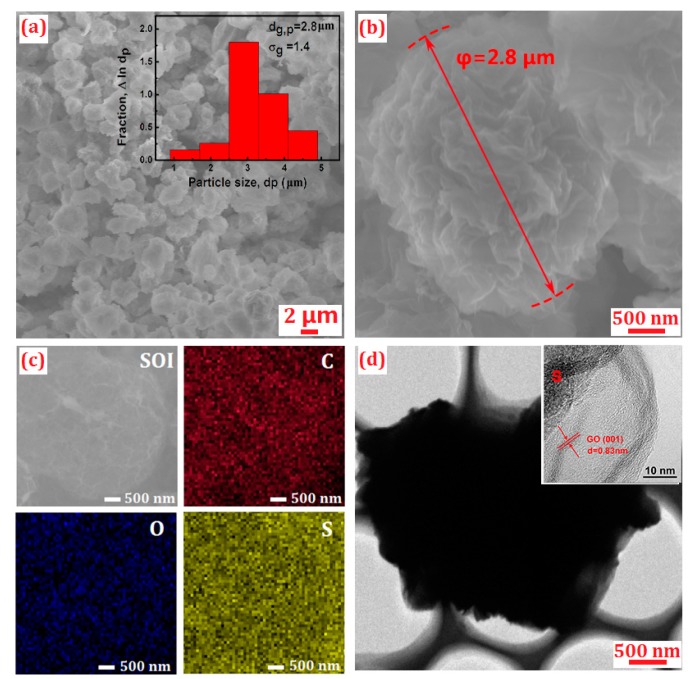
(**a**,**b**) SEM images of S/GO composites and the particle-size distribution of S/GO composites; (**c**) EDS mapping showing the distribution of carbon (C), oxide (O), and sulfur (S), in the S/GO composites; (**d**) TEM images of S/GO composites at different magnifications.

The SEM images of the S/GO composite powders are shown in Figure 3a,b. Figure 3a shows that the S/GO composite powders mainly consisted of homogeneous and discrete spherical micro-particles, although a small number of clusters were also detected. The particle-size distribution of the S/GO composite is observed in the inset of Figure 3a. The geometric mean diameter (*d*_g,p_ = 2.8 μm) of the particles, and the geometric standard deviation (σ_g_ = 1.4), were obtained. As shown in Figure 3b, at a higher magnification, the SEM image revealed a large number of wrinkles on the surface of the S/GO composites. These wrinkles provide space to accommodate the volume expansion of sulfur during the charging and discharging process. This speculation was further confirmed by the TEM images. As shown in Figure 3d, the black areas (sulfur) were homogenously distributed throughout the material. The TEM also showed that the sulfur was encapsulated by the GO material, which is shown as a layer on the surface of the composites. The particular architecture of the cathode material enhances its electrical conductivity, thus improving the discharge capacity and cycling performance of the lithium sulfur battery. In accordance with the above XRD analysis, crystal lattice fringes of sulfur were observed more clearly using HRTEM, as shown in Figure 3d, which indicates that the sulfur nanoparticles were effectively mixed with GO. The high-resolution TEM image exhibited clear lattice fringes, as shown in Figure 3d, with a distance of 0.83 nm, and could be indexed to the (001) crystal plane of the GO phase.

The energy dispersive X-ray spectroscopy (EDS) elemental mapping results confirmed a uniform distribution of sulfur on the surface of the GO composite powders, and the dispersion of C, O, and S, in S/GO composite powders was tested. The C (red), O (blue), and S (yellow) boxes, in Figure 3c, show the element mapping images of the C and S elements in the S/GO composite nanosphere. This indicates that sulfur nanoparticles are uniformly dispersed in the S/GO composite powders.

**Figure 4:**
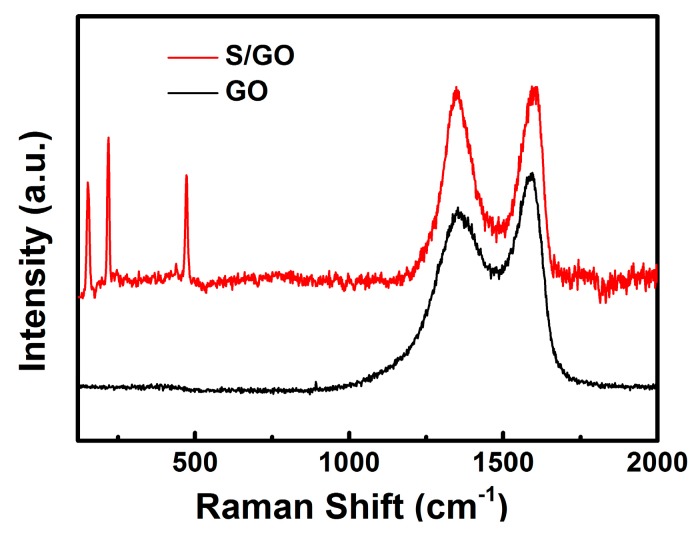
Raman spectra of GO and S/GO composite powders.

The Raman spectrum of the GO and S/GO composite powders is shown in Figure 4. Three peaks of S/GO composite powders, at 472, 219 and 151 cm^−1^, indicate the S–S bond in the composite. At the same time, it is clear that both the GO and S/GO composite powders show main peaks at 1353 cm^−1^ and 1592 cm^−1^, which are the characteristic peaks of the D and G carbon bands, respectively. The D band is a characteristic peak of defect sites and disorders [22], and the G band is the graphene-related E_2g_ phonon at the Brillouin zone center [23,24]. In this case, the intensity ratio of the D/G bands (*I*_D_/*I*_G_) for the S/GO composite powders is 0.90, which is larger than for GO (0.85). It is known that *I*_D_/*I*_G_ varies inversely with crystal size [25,26], suggesting that an increase in the *I*_D_/*I*_G_ value is related to a decrease in crystal size which, in turn, indicates that there is high sulfur dispersion. The Raman spectrum can be used to explain the significant structural changes that occur during the chemical process from GO to S/GO composite powders.

**Figure 5:**
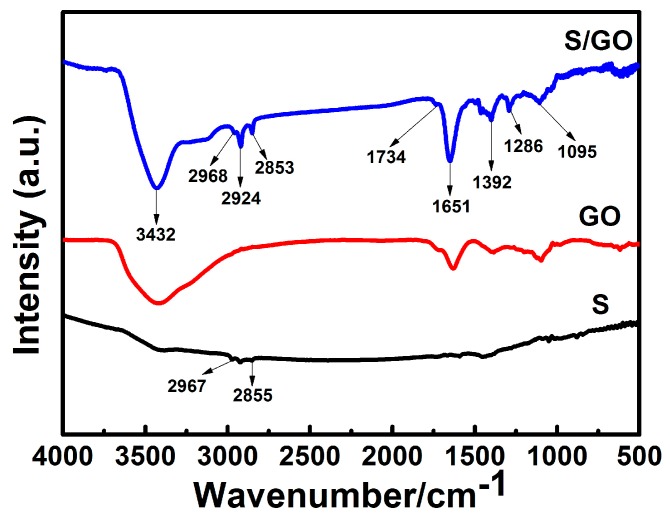
FT-IR spectra of S, GO, and S/GO composite powders.

An FT-IR spectrometer was used to investigate the surface functional groups of S/GO composite powders, GO and S, as shown in Figure 5. A large number of functional groups appeared in the FT-IR spectrum of the S/GO composite. As shown in the FT-IR spectrum of the S/GO composite powders in Figure 5, the peak that appears at 3432 cm^−1^ corresponds to –OH bond stretching vibrations in the hydroxyl group, and the peak at 1734 cm^−1^ corresponds to C=O bond stretching vibrations in the carbonyl group. Simultaneously, the stretching vibration peaks that corresponded to the carbonyl C–O (1286 cm^−1^) and the epoxy –C–O (1095 cm^−1^) still existed. The CH_2_ group stretching vibrations were at 2968 cm^−1^ and 2853 cm^−1^, and the C=C stretching vibrations of aromatic rings were at 1651 cm^−1^ and 1392 cm^−1^. Sulfur atoms existed in the form of disulfide bonds between 2855 cm^−1^ and 2967 cm^−1^. At the same time, the characteristic disulfide bond peaks located in the FT-IR spectra of S/GO suggest that a hydrogen bond was formed between carbonyl and sulfonic groups. 

**Figure 6:**
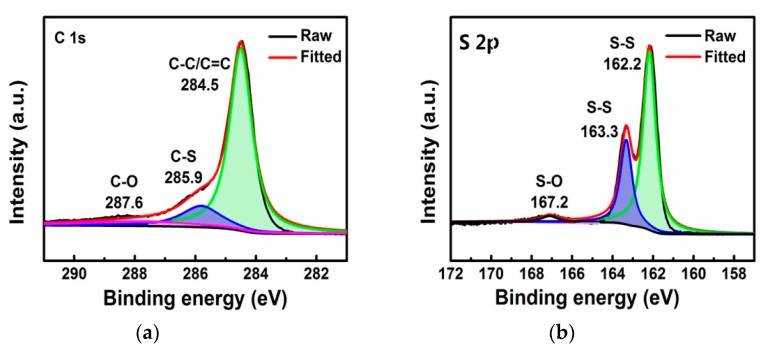
XPS spectra of S/GO for (**a**) C 1s and (**b**) S 2p peaks.

The various functional groups and elemental compositions of S/GO composite powders were further studied using XPS. As shown in Figure 6a, the characteristic peak at 284.5 eV in the C1s spectra for the S/GO composite powders belongs to carbon sp^2^ hybridization, namely C–C or C=C, and the characteristic peak of C–O is at 287.6 eV. The weak peak proves that the S/GO composite powders have oxygen-containing functional groups. The XPS C1s spectra of S/GO composite powders confirm the presence of a distinct peak (285.9 eV) corresponding to the C–S bonds, which indicates that certain chemical bonds exist between S and GO in the S/GO composite. The absence of the O–C=O bonding in the S/GO composite powders indicates that the edge sites of the S/GO composite powders are fully functionalized by S nanoparticles [27]. The S 2p corresponding XPS spectra of the S/GO composite powders showed two peaks of S 2p3/2 and S 2p1/2 components located at 162.2 eV and 163.3 eV, as shown in Figure 6b, which is the characteristic peak of solid sulfur in composites [28]. The peak of 167.2 eV, as shown in Figure 6b, corresponds to the surface oxidation of S or the interaction between S and GO [29]. Consequently, we believe that the results from the XPS analysis provide strong evidence that sulfur was formed on GO. The XPS results for the S/GO composite powders were well supported by the FT-IR results presented in Figure 6b. The characterization done by XRD, XPS, Raman, and FT-IR, suggests that the synthesis of GO by chemical and spray drying methods, along with sulfur composites, was successful.

**Figure 7:**
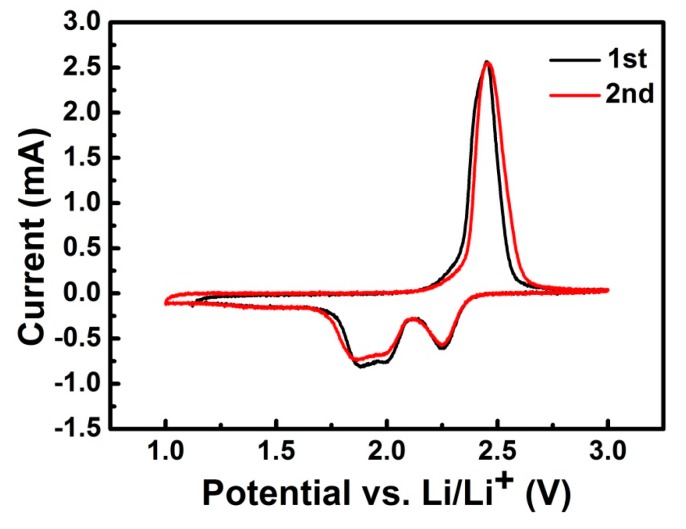
CV profiles of lithium sulfur batteries with the S/GO composite powders (scan rate of 0.1 mV s^−1^).

The S/GO composite powders serving as cathode materials were assembled in CR2025 cells, and electrochemical performance was detected at room temperature. The cyclic voltammogram (CV) curves of the S/GO composite powders were recorded at the scanning rate of 0.1 mV s^−1^ in a voltage window between 1.0 V and 3.0 V. In the first cycle, two obvious reduction peaks and one sharp oxidation peak were detected, respectively, as shown in Figure 7. These two reduction peaks were related to two different electrochemical reactions between sulfur and lithium. The upper peak of ~2.25 V was due to the multistep electrochemical reduction of S into high-order lithium polysulfides (Li_2_S*_n_*, 4 < *n* < 8), while the lower peaks, located at 2.04 and 1.87 V, might represent further reduction into Li_2_S_2_ or Li_2_S, respectively [30,31]. The oxidation peak observed at 2.4 V corresponds to the oxidation process of Li_2_S [32]. The CV curves after two cycles overlapped considerably, with the first cycle’s CV curve showing no obvious change. This indicates good cycling stability due to the nano-sized network structure of the S/GO composite powders, which could provide the very short diffusion route of the charge carriers and alleviate the volume expansion of the electrodes.

**Figure 8:**
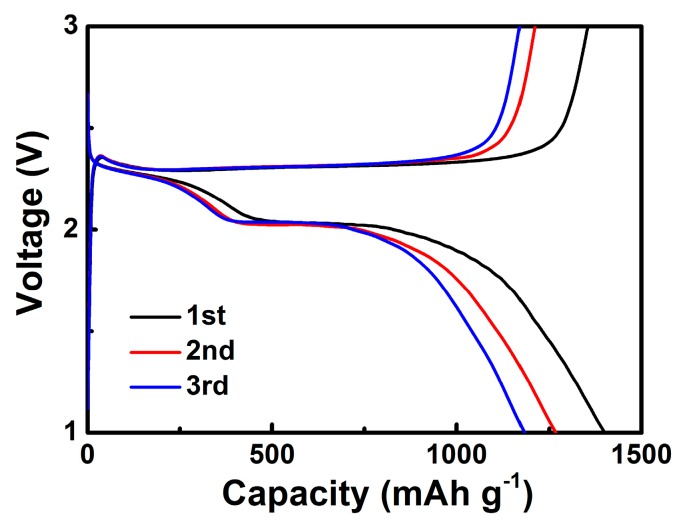
Charge and discharge curves of the S/GO composite electrode.

Figure 8 shows the galvanostatic charge/discharge profiles of the S/GO composite powders at 0.1 C in the potential range of 1.0 V to 3.0 V. There were two plateaus corresponding to the reaction between lithium and sulfur, which is in accordance with CV behaviors. The very short potential plateau (~2.35 V) was related to the formation of soluble lithium polysulfides in electrolytes. The other was a larger potential plateau (~2.05 V) and indicated that the reaction kinetics were lower than the formation of lithium polysulfides [33,34,35].

**Figure 9:**
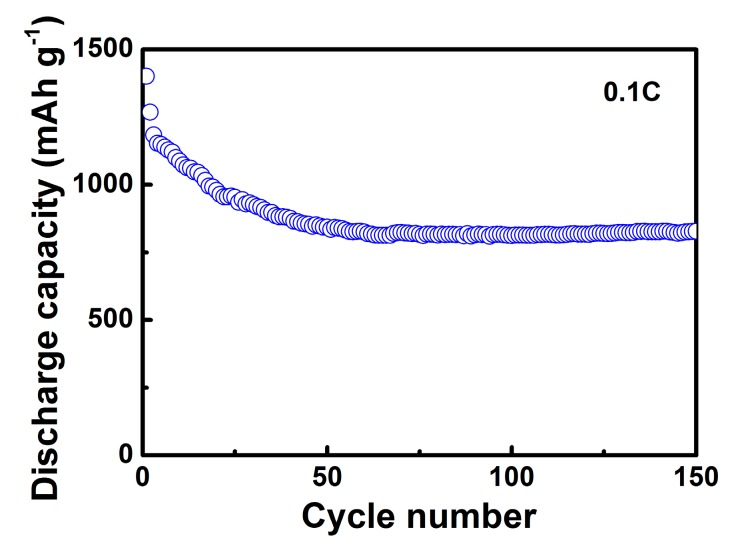
Cycling performance of the S/GO composite electrode at 0.1 C.

As shown in Figure 9, the initial discharge capacity of the S/GO composite powders was measured to be as high as 1400 mAh g^−1^. The initial reversible discharge capacity of the S/GO composite powders, prepared from the spray drying method, was 1268 mAh g^−1^. After 150 cycles, the discharge capacity of the S/GO composite powders gradually decreased and maintained 828 mAh g^−1^, which represents high capacity retention of 65%. Furthermore, the coulombic efficiency of the S/GO composite cathode approached 100%, implying perfect control of the shuttle effect.

**Figure 10:**
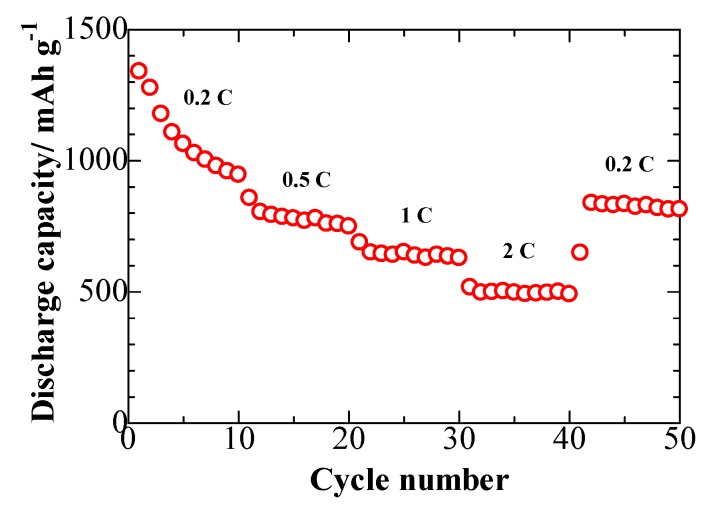
Rate performance of the S/GO composite electrode.

Figure 10 presents the rate performance of the S/GO composite electrode. The average discharge capacities of 1029, 772, 645, and 498 mAh g^−1^, were achieved at current densities of 0.2 C, 0.5 C, 1 C, and 2 C, respectively. Moreover, when the current density was switched back to 0.2 C, the S/GO composite cathode could recover its capacity, delivering 835 mAh g^−1^. This excellent rate performance of the cathode could be ascribed to its unique, wrinkled, microspherical architecture with internal void space agglomerates, which create pathways for electrolyte and Li-ion transport, leading to the enhanced activity of the composite.

**Figure 11:**
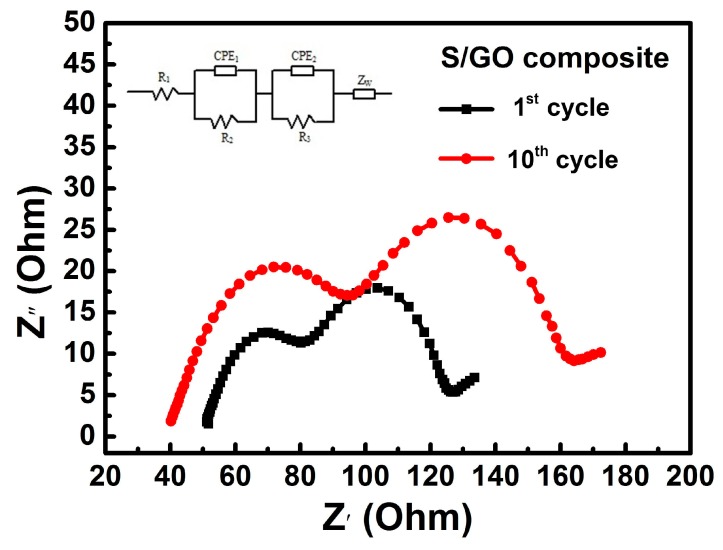
Electrochemical impedance spectroscopy (EIS) impedance of a lithium sulfur battery with the S/GO composite (inset shows the obtained equivalent circuit).

The electrochemical impedance spectroscopy (EIS) measurement was also tested between the frequency range of 0.01 Hz to 100 kHz. Figure 11 exhibits the EIS results of the S/GO composite powders at the end of the 1st and 10th discharge stage. The EIS spectra were composed of two depressed semicircles in the high and medium frequency regions, respectively, and a decline line in the low frequency region. The high frequency region of the semicircle was associated with the charge transfer resistance of lithium polysulfides, while the medium frequency region of the semicircle referred to the formation of Li_2_S or Li_2_S_2_ [36,37]. Because of the ohmic resistance of the electrolyte, separator, and electrical contacts, the EIS spectra intercepted the real axis in the high frequency region. During the charge–discharge cycle process, Li_2_S or Li_2_S_2_ can form on the elemental sulfur surface, preventing further dissolution into the electrolyte and, therefore, improving interfacial resistance, which is reflected by the larger medium frequency semicircle at 10 cycles [38,39].

The inset in Figure 11 is a simple equivalent circuit model applied to fit the EIS, where *R*_1_ represents the combined internal ohmic resistance of the electrolyte, separator, and electrode interfaces; *R*_2_ represents the charge transfer resistance of the electrode interfaces; and *R*_3_ represents the interfacial resistance of the reduction products of Li_2_S_2_ or Li_2_S. *R*_2_ and *R*_3_ gradually increase by the 10th cycle, which indicates an increase in charge transfer and interfacial resistance, respectively. The interfaces were stabilized during discharge–charge cycles, which could be why the electrochemical performance stabilized.

When compared to other related work (Table 1), our S/GO composite exhibited superior electrochemical performance [40,41,42,43,44,45]. The results indicated that the homogeneous distribution of wrinkled spherical sulfur micro-particles in the graphene oxide conductive network enhanced effective electrolyte contact and increased the reaction area, improving the discharge capacity and cycling performance of the lithium sulfur batteries.

## 3. Materials and Methods

### 3.1. Material Preparation

**Figure 12:**
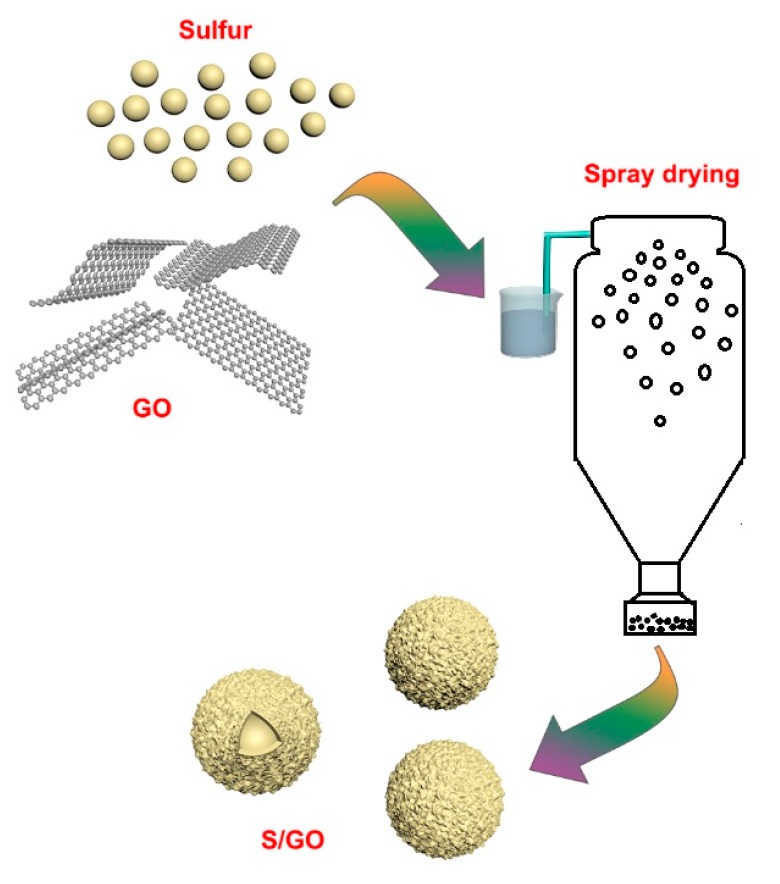
Schematic diagram of the fabrication of the S/GO composite powders.

GO was synthesized from natural graphene using the optimized Hummers method. The prepared graphene oxide aqueous solution was separated, thoroughly rinsed with deionized water by sonication five times, and graphite oxide dispersion (2 mg mL^−1^) was obtained. The nano-sulfur aqueous suspension (3 mg mL^−1^) was prepared with commercial nano-sulfur powder, purchased from Shanghai Huzheng Nano Technology Co., Ltd., Shanghai, China. The synthesis procedure of the sulfur/graphite oxide (S/GO) composite powders is shown in Figure 12. The composite was formed by adding the sulfur nanoparticle dispersion to the GO dispersion, dropwise, while mixing it with a thermostatic magnetic mixer (Zhengzhou Teer Instruments, Zhengzhou, China). The mixture was sonicated using an ultrasonic cell disruptor (Ningbo Scientz Biotechnology Co., Ningbo, China) for 1 h and 30 min to form a uniform suspension. The homogenous solution was further spray dried at 200 °C to obtain the S/GO composite precursors, with a flow rate of 8 mL min^−1^. After spraying, the as-prepared composite was dried in a vacuum at 60 °C for 12 h.

### 3.2. Characterizations

The crystal structural characterizations of the sample were characterized using an X-ray diffraction (XRD) system (D8 Discover, Bruker, Karlsruhe, Germany), with Cu Kα radiation (λ = 1.542 Å), at a working voltage of 40 kV, between 10° and 70° room temperature. The morphology and microstructure of samples were exhibited by scanning electron microscopy (SEM, JSM-6700F, JEOL, Tokyo, Japan), coupled with EDS analysis and high-resolution transmission electron microscopy (HRTEM, JEM-2100F, JEOL, Tokyo, Japan), respectively. The Raman spectra were obtained on a DXR Raman imaging microscope (Thermo Scientific, Waltham, MA, USA) system using a laser excitation wavelength of 514 nm. The chemical transformation of the samples was investigated by Fourier Transform Infrared Spectroscopy (FT-IR, Nicolet, Thermo Scientific, Waltham, MA, USA). X-ray photoelectron spectroscopy (XPS) data were obtained with a PHI 5000 Versa Probe system (Ulvac-Phi, Kanagawa, Japan).

### 3.3. Electrochemical Measurements

The microporous polyethylene membrane, which served as the separator, was soaked in the electrolyte, which was a solution of 1 M lithium trifluoromethanesulfonate (LiCF_3_SO_3_) mixed with 1,2-dimethoxy ethane and 1,3-dioxolane (1:1 by volume). The working electrode was prepared by mixing 80 wt % S/GO composite powders as active materials, polyvinylidene fluoride as a binder, and 10 wt % acetylene black conducting agent dissolved in *N*-methylpyrrolidone. The resultant slurry was uniformly coated onto aluminum foil and dried in a vacuum at 60 °C for 12 h. The active material was around 2 mg cm^−2^. The electrochemical performances of the S/GO composite were assembled in a high purity argon-filled MBraun glove box, with a lithium foil as the counter electrode, using CR2025 cells. The cells were tested using Galvano-static on a multichannel battery tester (BT-2000, Arbin Instruments, College Station, TX, USA), from 1.0 and 3.0 V vs. Li/Li^+^. All electrochemical measurements were performed at room temperature.

## 4. Conclusions

In summary, the functional groups of GO prevent the dissolution of polysulfides from electrodes. The excellent electrochemical performances can be ascribed to the wrinkled micro-spherical structure of S/GO composite, in which, GO works as a host to confine sulfur and provides enough space to accommodate sulfur and polysulfides, helping to hinder the dissolution of the active materials and suppress the migration of polysulfide ions. This work also proved that sulfur-nanoparticles could coat onto graphene oxide uniformly via spray drying, enhancing electrical conductivity and alleviating the shuttle effect. It can be seen that the S/GO composite powders as cathode materials allow lithium sulfur batteries to obtain a high level of performance.

## Figures and Tables

**Table 1:** Literature comparison of the electrochemical performances of composite cathode materials for lithium sulfur batteries.

Cathode	Cycle Number	Capacity Remaining (mAh g^−1^)	Current Density	Reference
S/rGO	20	592.4	0.2 mA cm^−2^	[38]
S/GO	160	620	0.1 C	[39]
S/rGO	100	338	0.2 C	[40]
S/rGO	150	600	2 C	[41]
S/3DNG	500	578	0.5 C	[42]
S/G	200	363	1 C	[43]
